# Spontaneous pneumothorax, pneumomediastinum and subcutaneous emphysema in non-ventilated COVID-19 patients

**DOI:** 10.2144/fsoa-2021-0090

**Published:** 2021-11-18

**Authors:** Shadi Hamouri, Mohammad AlQudah, Omar Albawaih, Nabil Al-zoubi, Sebawe Syaj

**Affiliations:** 1 Department of General Surgery & Urology, Faculty of Medicine, Jordan University of Science & Technology, King Abdullah University Hospital, Irbid, 22110, Jordan; 2 Department of Pathology & Microbiology, Faculty of Medicine, Jordan University of Science & Technology, Irbid, 22110, Jordan

**Keywords:** COVID-19, pneumomediastinum, spontaneous pneumothorax, subcutaneous emphysema

## Abstract

**Aim:**

Pneumothorax (PNX), pneumomediastinum (PMD) and subcutaneous emphysema (SCE) are COVID-19 complications related to positive-pressure ventilation. We analyzed the pathophysiology of these complications without ventilation.

**Patients & methods:**

Out of 1845 admitted COVID-19 patients, we retrospectively collected data for 15 patients, from a tertiary medical center, from 1 October 2020 to 31 March 2021.

**Results:**

Five patients suffered from spontaneous PNX, 8/15 developed PMD and 8/15 developed SCE. The mean BMI was 29.7, as most patients were obese or overweight. Most patients had lymphocytopenia and increased C-reactive protein, ferritin and lactate dehydrogenase levels. Eleven patients succumbed to the disease.

**Conclusion:**

Risk factors of spontaneous PNX, PMD and SCE in COVID-19 patients need further investigations by conducting more comprehensive case–control studies.

## Lay abstract

We have investigated spontaneous alveolar rupture as a complication in 15 COVID-19 patients. Manifested as pneumothorax, pneumomediastinum and subcutaneous emphysema, these complications are less common in patients without mechanical ventilation. Management of these patients was either conservative or by insertion of a chest tube. Eventually, 11 out of 15 patients have passed away due to respiratory failure.

## Graphical abstract

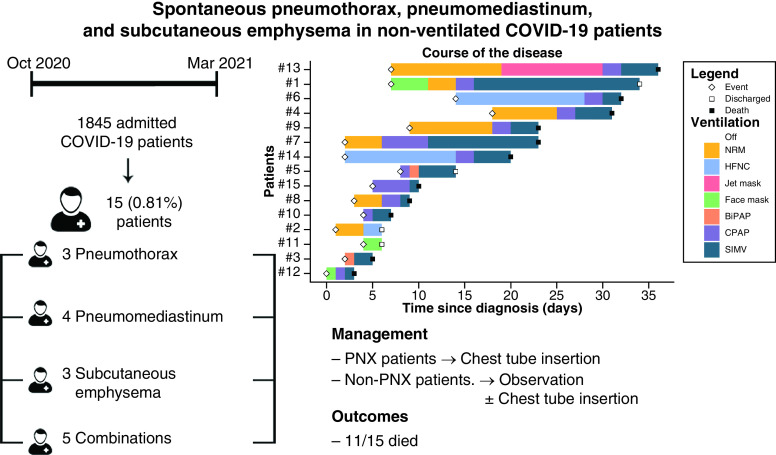

In 2019, an outbreak was caused in Wuhan, China, by SARS-CoV-2. It soon became a pandemic and a global health issue [1,2]. COVID-19 is the leading cause of admission of patients to the intensive care unit, with a potential for fatality, especially for elderly patients more than 65 years of age and patients with comorbidities including obesity, hypertension, congestive heart failure, diabetes, asthma, chronic kidney disease and those who are immune depressed, including patients with cancer [3]. Death is due mainly to acute respiratory distress syndrome (ARDS) and, eventually, acute respiratory failure [4]. In addition, around 15–20% of patients require intensive care and ventilatory support [5], with an increasing number of complications related to positive-pressure ventilation such as pneumothorax (PNX), pneumomediastinum (PMD) and subcutaneous emphysema (SCE), all collectively termed as pulmonary barotrauma [6].

Pulmonary barotrauma is characterized by extra-alveolar air due to rupture of alveolar walls and air leakage to the perivascular sheath [7]. Two reports found that 24% of mechanically ventilated COVID-19 patients suffered from PNX or PMD [8,9]. Interestingly, some COVID-19 patients spontaneously develop PNX, PMD and SCE without intubation or positive-pressure ventilation. This unique finding has been recently reported in some rare cases in the literature [10–12]. However, more investigation is required about the clinical features and the pathophysiology of COVID-19 in such complications. Therefore, this study presents a series of cases that experienced spontaneous PNX, PMD and SCE and their clinical characteristics, laboratory results and outcomes.

## Methods

### Study design & patients

We present a retrospective case series in which all patients were consecutive cases, hospitalized at our tertiary medical center from 1 October 2020 to 31 March 2021. The diagnosis of COVID-19 was made using a real-time reverse transcriptase-PCR test, executed on a nasopharyngeal swab. Patients of all ages were included, and patients receiving positive-pressure ventilation at the time of presentation with extra-alveolar air were excluded. Out of 1845 COVID-19 patients hospitalized at our institution, we describe 15 cases of spontaneous PNX, PMD and SCE, or a combination of these pathologies.

### Patient management

The management protocol of patients admitted to the clinical wards include simple oxygen (O_2_) therapy (nasal cannula/O_2_ facemask/nonrebreather mask [NRM]), high-flow nasal cannula, dexamethasone 6 mg Q24 IV, prophylactic low-molecular weight heparin and proton pump inhibitors. Remdesivir was also included in the protocol and given according to the guidelines. Spontaneous PNX, PMD and SCE were diagnosed by clinical evidence of the pathology and confirmed by chest x-ray. Computed tomography (CT) scan of the chest was not part of our investigation protocol. However, it has been performed in selected patients for specific indications such as the suspicion of pulmonary embolism. We have adopted the following strategies regarding the patient’s treatment: Patients who presented with isolated PNX with or without PMD or SCE were treated with chest tube insertion. The size of the tubes ranged from 20 to 24 French. Patients who presented with isolated PMD or SCE were observed. Chest tubes were inserted in this group of patients only if they developed PNX during the illness.

Patients who have progressed to severe disease according to the National Institutes of Health severity criteria [14] eventually were put on positive-pressure ventilation, either noninvasive ventilation like continuous positive airway pressure (CPAP)/bi-level positive airway pressure or invasive mechanical ventilation (IMV) by endotracheal intubation.

### Data collection

We retrospectively extracted clinical data, including presenting symptoms, comorbidities, BMI, type of event (PNX, PMD, SCE), the timing of the event, arterial blood gases, laboratory values, anticoagulant and steroid treatment, mode and timing of postevent ventilation, mortality and length of hospital stay. BMI was defined according to the WHO as underweight (BMI ≤18.5), normal (BMI: 18.5–24.9), overweight (BMI: 25–29.9) and obese (BMI ≥30) [15].

### Statistical analysis

We used R statistical language (version 4.0.5) to create figures, generate summary measures, including means for continuous variables, frequencies and percentages for categorical variables [16]. In addition, tables were used to embed individual patient data.

## Results

### Demographics, comorbidities & presentation

We evaluated 15 COVID-19 cases in this case series who suffered spontaneous PNX, PMD or SCE. The sample contained 12 males and three females, their ages ranged from 21 to 77 years, and 7/15 of patients were active smokers. Eight patients were obese, six were overweight, one patient fell within the normal range for weight and the overall mean BMI was 29.7. Five patients had hypertension, three had diabetes mellitus, five suffered from ischemic heart disease and none suffered from heart failure or chronic kidney disease. Chronic obstructive pulmonary disease (COPD) was found in four patients, one patient had asthma and one patient suffered from non-small cell lung cancer. None of the patients had interstitial lung disease. Most patients presented with fever (14/15), dry cough (13/15) and shortness of breath (12/15). Chest pain was present in 3/15 patients, and 2/15 patients had mild hemoptysis. Patients were treated with simple face mask O_2_ therapy, NRM and high-flow nasal cannula. Patients’ characteristics and management are summarized in Table 1.

**Table 1.** Patients’ clinical characteristics.

Patient	Age (years)	Gender	BMI	Smoking	Comorbidities	Lung diseases	Symptoms	Event type	Chest tube side	O_2_ therapy	LOS	Day of event	Outcome
1	21	F	26	No	-	-	Fever, dry cough	PNX, PMD, SCE	Bilateral	NRM	19	7	Discharged
2	51	M	29.7	No	DM, IHD	-	Fever, SOB, dry cough	PNX	Left	Face mask	9	1	Discharged
3	77	M	23	Yes	HTN	COPD, NSCLC	SOB, dry cough, hemoptysis	PNX	-	NRM	3	2	Dead
4	67	M	31	Yes	HTN, DM, IHD	-	Fever, dry cough, chest pain, hemoptysis	SCE	-	NRM	29	18	Dead
5	58	M	30	No	-	-	Fever, SOB	PMD, SCE	Right	NRM	15	8	Discharged
6	62	M	28	Yes	-	COPD	Fever, SOB, dry cough	PMD		NRM	26	14	Dead
7	49	F	28	No	-	COPD, Asthma	Fever, SOB, dry cough, chest pain	PMD, SCE	Bilateral	Face mask	5	2	Dead
8	68	M	33	Yes	-	-	Fever, SOB, dry cough	PMD, SCE	Right	Face mask	6	3	Dead
9	62	M	32.5	Yes	HTN, IHD	-	Fever, dry cough	SCE	Left	NRM	14	9	Dead
10	65	M	34	No	HTN, DM, IHD	-	Fever, SOB, dry cough	PMD	Left	Face mask	5	4	Dead
11	26	M	32	No	-	-	Fever, SOB, dry cough	PMD	-	HFNC	4	4	Discharged
12	34	F	30	No	-	-	Fever, SOB, chest pain	PNX, SCE	Left	Face mask	2	1	Dead
13	41	M	26.5	Yes	-	-	Fever, SOB	PMD	-	NRM	31	7	Dead
14	66	M	26.9	Yes	HTN, IHD	COPD	Fever, SOB, dry cough	PNX	Left	NRM	17	2	Dead
15	53	M	34.6	No	-	-	SOB, dry cough, chest pain	SCE	-	NRM	5	5	Dead

COPD: Chronic obstructive pulmonary disease; DM: Diabetes mellitus; HFNC: High-flow nasal cannula; HTN: Hypertension; IHD: Ischemic heart disease; LOS: Length of stay; NRM: Nonrebreather mask; NSCLC: Non-small cell lung cancer; PMD: Pneumomediastinum; PNX: Pneumothorax; SCE: Subcutaneous emphysema; SOB: Shortness of breath.

### Laboratory investigations & imaging

**Figure 1.**
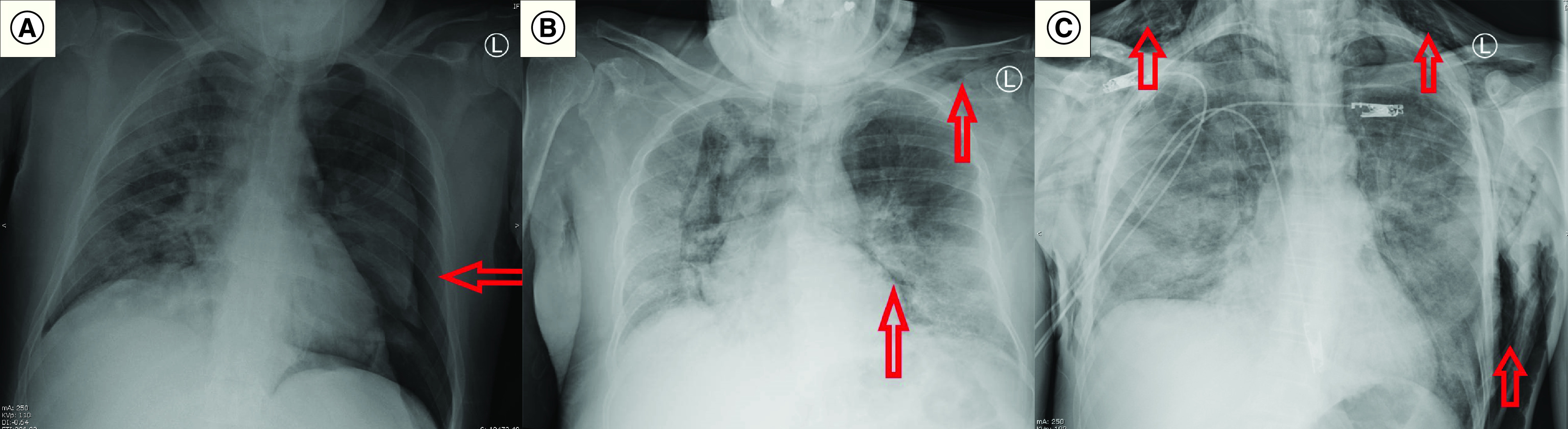
Radiological findings of pneumothorax, pneumomediastinum and subcutaneous emphysema. X-ray images showing: **(A)** PNX in Case #2. **(B)** PMD and SCE in Case #5. **(C)** SCE in Case #15. PMD: Pneumomediastinum; PNX: Pneumothorax; SCE: Subcutaneous emphysema.

**Figure 2.**
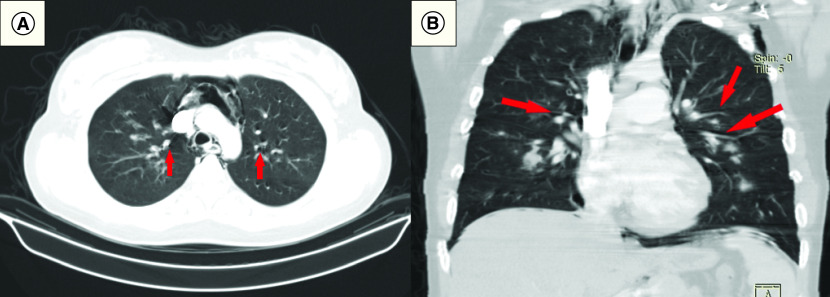
Radiological findings of pneumothorax, pneumomediastinum and subcutaneous emphysema. Computed tomography images demonstrating PMD and Macklin’s effect in: **(A)** Axial section. **(B)** A coronal section. PMD: Pneumomediastinum.

Upon admission, laboratory tests and radiological imaging were ordered for all patients (Table 2). C-reactive protein (CRP), ferritin and lactate dehydrogenase measures were elevated in most patients. Lymphocyte counts were low in most patients, only four patients had thrombocytopenia and d-dimer was positive in seven patients. Laboratory values are shown in Supplementary Table 1. Chest x-rays showed in addition to the PNX, PMD and SCE (Figure 1A–C), bilateral infiltrates in 11 patients (6: diffuse; 5: lower zones), left lung involvement in two patients (diffuse and upper zone) and diffuse in the right lung in two patients. Only two patients underwent thoracic CT images, and Macklin’s effect of air dissection along bronchoalveolar sheaths was found in both images (Figure 2A & B).

**Table 2.** Laboratory and radiological workup.

Patient	CRP	CK	Fr	U	Cr	LDH	Troponin	WBC	Hb	Plt	N	L	DD	CXR findings
1	↑	N	N	↓	↓	↑	N	N	↓	↓	N	↓	↑	Bilateral lower
2	↑	-	-	↓	N	↑	-	N	↓	N	N	↓	N	Bilateral lower
3	↑	N	↑	N	↓	-	N	↓	N	N	N	↓	N	Bilateral diffuse
4	↑	N	↑	N	N	↑	N	N	N	N	↑	↓	↑	Bilateral diffuse
5	↑	↑	↑	N	N	↑	N	N	↓	N	N	↓	↑	Bilateral diffuse
6	↑	N	↑	N	N	↑	N	N	↓	↓	↑	↓	↑	Bilateral diffuse
7	↑	N	↑	↓	N	↑	N	↑	N	N	↑	↓	N	Right diffuse
8	↑	↑	↑	N	N	↑	N	N	↓	N	N	↓	N	Bilateral lower
9	-	N	↑	↓	N	↑	N	N	↓	N	N	↓	↑	Bilateral diffuse
10	-	N	-	N	N	↑	N	N	↓	N	↑	↓	N	Bilateral lower
11	↑	↑	↑	N	↓	↑	-	↑	N	N	↑	↓	↑	Left upper
12	↑	-	-	↓	N	↑	-	N	↓	↓	N	↓	N	Bilateral diffuse
13	-	-	-	N	N	↑	-	↓	↓	↓	↓	↓	N	Left diffuse
14	↑	↑	↑	N	↑	↑	N	N	↓	N	N	N	↑	Right diffuse
15	↑	↑	↑	↓	N	↑	-	↑	↓	N	↑	↑	↑	Bilateral lower

CK: Creatinine kinase; Cr: Serum creatinine; CRP: C-reactive protein; CXR: Chest x-ray; DD: d-dimer; Fr: Ferritin; Hb: Hemoglobin; L: Lymphocyte; LDH: Lactate dehydrogenase; N: Neutrophils; Plt: Platelet; U: Urea; WBC: White blood cell.

### Treatment

Although none of the patients were ventilated, they developed spontaneous PNX (5/15), PMD (8/15) or SCE (8/15). While most of the patients had either PNX, PMD or SCE, only three patients had both PMD and SCE, one had both PNX and SCE, and one patient had all three complications combined (Table 1). Days from diagnosis until the event ranged from 1 to 18 days (median: 4 days). Management of injury was by inserting a chest tube for nine patients (five left, two right and two bilateral). All except for one patient was managed with a chest tube that was 20–24 French in size. The exception was one 28-French tube. The rest of the patients were treated conservatively. We noticed complete resolution of the PNX immediately after insertion of the chest tube without evidence of air leak except in Case # 1, who developed prolonged air leak for 13 days and then stopped spontaneously. No patient has required an ipsilateral second chest tube insertion. The median hospital stay was 9 days (range: 2–31 days). Thirteen patients were treated with steroids, and 13 patients were treated with prophylactic low-molecular weight heparin anticoagulants.

**Figure 3.**
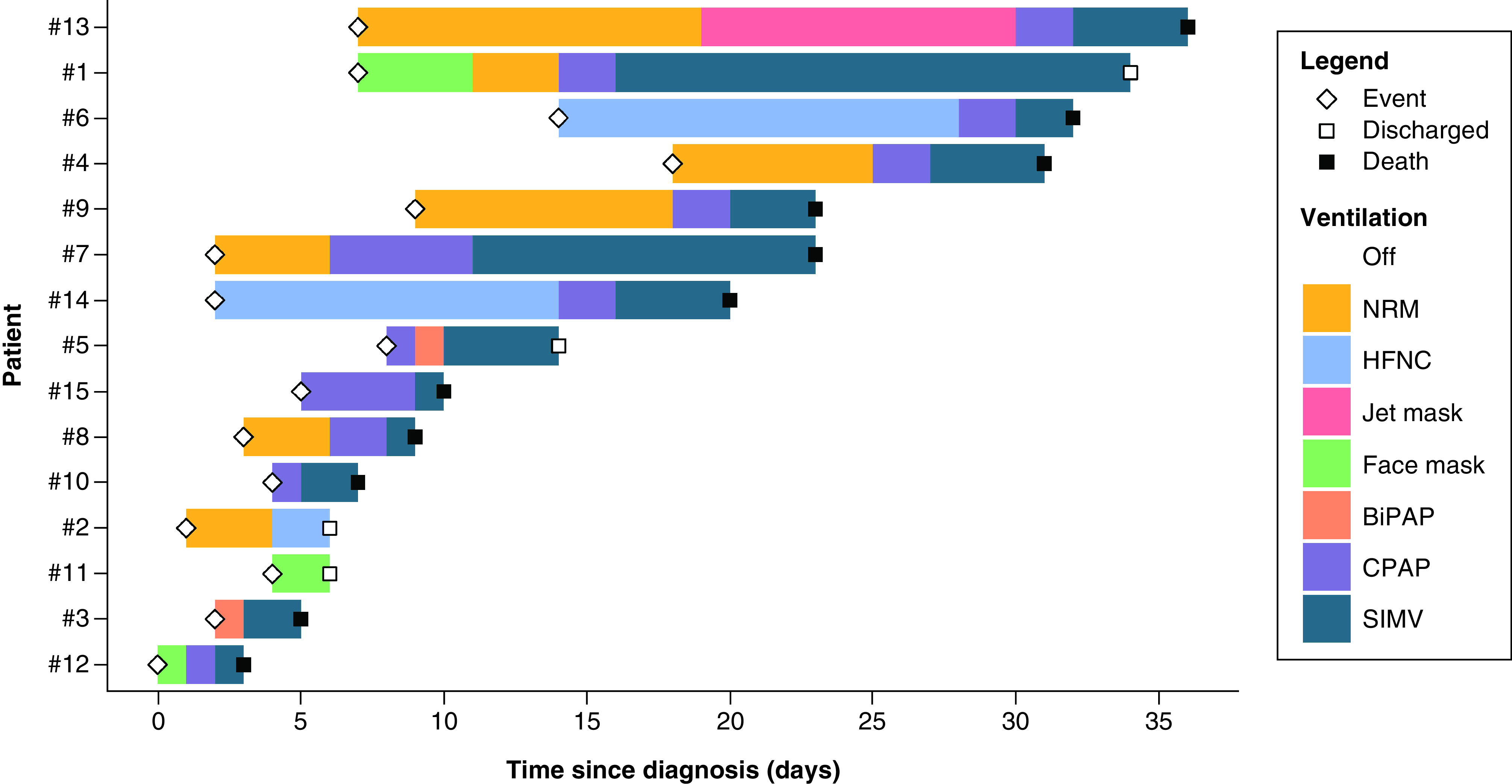
A swimmer’s plot displaying the timeline of the ventilation series for each case, including the timing of the injury (diamond), discharge (white square) and death (black square).

After the occurrence of the event, 12 patients were started on O_2_ therapy. Within a period ranging from 2 to 28 days, all patients required some sort of positive-pressure ventilation. They were started on noninvasive positive-pressure ventilation, including CPAP and bi-level positive airway pressure, and eventually intubation (Figure 3) (Table 3). Cases #5, #10 and #15 were started on CPAP directly after the injury. Unfortunately, 11 of the 15 patients have passed away due to disease progression. For PMD patients who got discharged (3/15), resolution of symptoms occurred in a period ranging from 8 to 30 days.

**Table 3.** Drugs and post event ventilation.

Patient	Steroids	Anticoagulants	Post-event ventilation	Days of post-event ventilation
1	-	LMWH	SIMV	10
2	Dexamethasone	LMWH + ASA	NRM	4
3	Methylprednisolone + Fluticasone	LMWH	SIMV	2
4	Dexamethasone	LMWH + ASA	SIMV	18
5	Dexamethasone	LMWH	SIMV	12
6	Methylprednisolone	LMWH	SIMV	14
7	Dexamethasone	LMWH	SIMV	2
8	Dexamethasone	LMWH	SIMV	3
9	Dexamethasone	LMWH + ASA	SIMV	9
10	Dexamethasone	LMWH + ASA	SIMV	4
11	Dexamethasone	LMWH	Face Mask	4
12	Prednisolone	-	SIMV	28
13	-	-	Jet ventilation	7
14	Dexamethasone + fluticasone	LMWH	SIMV	2
15	Dexamethasone	LMWH + ASA	SIMV	15

ASA: Acetylsalicylic acid; LMWH: Low-molecular weight heparin; NRM: Nonrebreather mask; SIMV: Synchronized intermittent mandatory ventilation.

## Discussion

This study presents a series of patients who manifested spontaneous alveolar rupture presenting as PNX, PMD and/or SCE. These injuries are considered relatively common complications for critically ill COVID-19 patients, mainly in mechanically ventilated patients [17,18]. However, our patients did not receive any positive-pressure ventilation before or at the time of the event. PNX was reported in 1.7% of SARS patients receiving positive-pressure ventilation [19]. At our institute, spontaneous alveolar injury prevalence was 15 out of 1845 (0.81%) COVID-19 pneumonia-hospitalized patients. This finding is comparable with the reported literature that does not exceed the prevalence of 1–2%, out of all COVID-19-hospitalized patients [9,10,12]. Tacconi *et al.* found that the crude incidence of PMD was higher in the second wave of COVID-19 with borderline significance. However, the difference gained statistical significance when subgrouping patients with non-IMV [20].

Other studies reported rare cases of such manifestations of COVID-19 [21–27]. Manna *et al.* reported a series of 11 patients who experienced spontaneous PMD and SCE with absence of or before intubation [21]. None of their patients were smokers, and they were predominantly overweight. Patients of our cohort were predominantly obese and overweight, and seven of them were active smokers. Cut *et al.* presented 11 similar cases, and all of them were nonsmokers. However, only three of them were obese [10]. Miro *et al.* investigated 40 spontaneous PNX patients in a case–control fashion. They found that complaining of shortness of breath and chest pain are significant predictors of spontaneous PNX in COVID-19 patients [28]. Twelve patients in our study had shortness of breath, but only three patients complained of chest pain.

The pathophysiology behind spontaneous lung injury is still under investigation, and several theories have been suggested, including but not limited to direct viral damage, microthrombosis, exaggerated immune response and obesity-related [12,27]. According to a histopathological postmortem study of 68 patients with COVID-19, grossly 92% of autopsies had lungs with increased weight (>1300 g). Diffuse alveolar damage was found in 87% of all cases, and large airway inflammation was found in 35/38 of autopsies that were never intubated. However, the authors have found no association between large airway inflammation and intubation. Seventy-one percent of these patients were on anticoagulants in that study, 88% of which had microthrombi of arterioles and capillaries [29].

As for special cases of COVID-19 with spontaneous PNX, PMD and SCE, Tucker *et al.* found increased lung weights, severe degree of alveolar and interstitial architectural damage in two autopsies of the nonintubated patients. Interestingly, they have found infarction in both patients and was acute hemorrhagic infarction in the second patient who underwent autopsy [27].

A systematic review including 63 studies of COVID-19 postmortem analyses suggested that diffuse alveolar damage is the most encountered histological finding, mainly in exudative and proliferative phases [30]. Diffuse alveolar damage is a hallmark of ARDS across different etiologies. It is characterized by permanent architectural damage of the alveolar-capillary barrier associated with a vascular pathology, which affects alveolar ventilation, causes hypoxemia and ultimately infarction of the tissue and cyst formation [29,31,32]. These circumstances could cause alveolar rupture, leading to dissection of air into the bronchovascular sheaths and spreading into the mediastinum, the so-called Macklin effect on CT images showing PMD [31], such as those found in two patients in our study who underwent CT scanning. In a meta-analysis, the overall prevalence of diffuse alveolar damage was found to be more common in SARS-CoV-1 (100.0%) than COVID-19 (80.9%) autopsies (p = 0.001). However, diffuse alveolar damage was the most common cause of death in both the groups [33].

In addition to the diffuse alveolar damage of pneumocytes and interstitium, SARS-CoV-2 affects pulmonary microcirculation by causing direct damage to vascular endothelial cells, leading to apoptosis, which eventually decreases the antithrombotic activity of the luminal surface. This endotheliopathy forming *in situ* microthrombi might be the main reason for pulmonary dysfunction [34]. Exposing endothelial tissue factor also leads to a state of hypercoagulability [35]. These microthrombi and platelet-fibrin thrombi resulting from diffuse alveolar damage affect both alveolar ventilation and perfusion by causing occlusion to microvessels, pulmonary tissue infarction and ultimately increasing the chance for air leak [27]. d-dimer is a crucial biomarker for monitoring the COVID-19 coagulation process and predicting thrombotic complications and critical illness [36,37]. Seven patients in our study had elevated d-dimer levels upon admission. Although 13/15 of our patients received anticoagulants, we suspect they experienced severe microthrombosis, leading their alveoli to necrose and rupture spontaneously.

Most patients had elevated inflammatory markers like CRP and ferritin, which are suggested predictors for COVID-19 severity [38–40], and are reported to correlate with d-dimer and IL-6 levels [41]. In addition, Miro *et al.* found that d-dimer and LDH levels were significant predictors of developing spontaneous PNX, but p-values were not significant after Bonferroni statistical correction [28]. Still, head-to-head pathological and serum comparisons between spontaneous PNX, PMD and SCE patients and control patients for COVID-19 are needed to confirm the effect of microthrombosis on alveolar rupture.

COVID-19 ARDS patients have a significantly higher BMI than non-COVID-19 ARDS patients [42]. Most patients in our series were either obese or overweight (14/15), which is highly associated with severe illness in COVID-19 patients according to a multicenter cohort in China. Wang *et al.*, having less than 5% of their patients on non-IMV, explained the mechanisms between obesity and overweight in contributing to COVID-19 severity through induction of chronic systemic inflammation via IL-6, IL-8 and TNF-α [43]. In another study, IL-6 was found to be 1.7-times higher in nonsurvivor COVID-19 patients than survivors [44]. As for lymphocytopenia, Tatum *et al.* found that neutrophil-to-lymphocyte ratio upon admission is a predictor of intubation in COVID-19 patients, two out of three of their patients were obese (mean BMI = 34.2), and they found that neutrophil-to-lymphocyte ratio significantly predicts disease mortality [45]. Miro *et al.* found that leukocytosis above 10 cells/μl was a significant predictor of spontaneous PNX. Conversely, obesity failed to predict spontaneous PNX [28].

The role of obesity in increasing the risk of thrombosis is not negligible, and its harmful effect on cardiorespiratory reserve should be considered as well. Because of these predisposing factors and others that are less understood, we must devote more effort to understanding the relationship of obesity’s contribution to the outcome of patients suffering from COVID-19 [43,46]. The mean BMI in our study was 29.7, which was higher than the mean BMI of 25.9 reported by Manna *et al.* for 11 patients. Four patients in this series already had COPD, and we hypothesize that COVID-19 further aggravated their injury leading to alveolar rupture. The outcome for these four patients was death due to respiratory failure.

We managed our patients by inserting chest tubes for nine patients and observing the other six patients. Ten patients in our study developed PMD and/or SCE without PNX; half were treated conservatively. Management of these patients is controversial; In a case series, Housman *et al.* found that conservative management is sufficient for SCE patients, even in massive sizes, unless there was a risk for developing PNX [47]. The same strategy worked for a mechanically-ventilated case with PMD and SCE [48]. On the contrary, a retrospective study of 122 patients suggests that thoracostomy tube insertion patients have better improvement than those treated conservatively [49]. Further research should be conducted to figure out the most suitable management plan for each scenario of barotrauma.

The age group is directly related to the COVID-19 fatality rate. A meta-analysis shows that the age-specific fatality rate is low for younger patients (around 0.01% at age 25) and becomes as high as 0.4% at age 55, 1.4% at age 65 and 4.6% at age 75. Around 72% of patients who passed away in our series were above the age of 50 [50].

Our study is limited by its retrospective nature of data collection. We did not run bleeding tests such as bleeding time, prothrombin time, partial thromboplastin time, fibrinogen or Von Willebrand factor or any viscoelastic testing to assess coagulation. Since our sample contained seven active smokers and four COPD patients, emphysema could have been a predisposing risk factor for alveolar rupture. Moreover, performing CT images was not part of our protocol, as it may detect subclinical PMD and SCE [51]. Lastly, since none of our patients underwent autopsy, we could not compare the existing literature on autopsy with our patients.

## Conclusion

Alveolar rupture manifested as PNX, PMD or SCE should be suspected in COVID-19 patients, spontaneously or more commonly in mechanically ventilated patients. Risk factors of spontaneous alveolar rupture and barotrauma need further investigation.

**Summary points**

- Spontaneous pneumothorax, pneumomediastinum and subcutaneous emphysema are rare complications that can occur without mechanical ventilation, with the prevalence of 0.81% of all COVID-19 patients.
- Several theories of COVID-19 pathophysiology in these complications have been proposed, but each requires extensive studies to confirm their validity.
- Autopsies of patients who experienced these phenomena are needed to discover the pathophysiology of alveolar rupture in COVID-19.
