# Supplementary material for: Spontaneous pneumothorax, pneumomediastinum and subcutaneous emphysema in non-ventilated COVID-19 patients
Source: Future Sci OA. 2021 Nov 18;8(2):FSO771. doi: 10.2144/fsoa-2021-0090 (PMC8609960; doi:10.2144/fsoa-2021-0090)
Supplement: Supplementary file 1 [file fsoa-08-771-s1.docx]

Table S1: Laboratory and Radiological workup

| Patient | CRP  (mg/L) | CK  (units/L) | Fr  (µg/L) | U  (mmol/L) | Cr  (mg/dL) | LDH  (units/L) | Troponin  (ng/L) | WBC  (per µL) | Hb  (g/dL) | Plt  (*10^3^ per µL) | N  (per µL) | L  (per µL) | DD  (ng/mL FEU) | CXR findings |
| --- | --- | --- | --- | --- | --- | --- | --- | --- | --- | --- | --- | --- | --- | --- |
| 1 | 73 | 51 | 24 | 0.9 | 32 | 402 | 0.021 | 5500 | 9.4 | 142 | 4785 | 511 | 1.26 | Bilateral Lower |
| 2 | 210 |  |  | 4.7 | 85 | 351 |  | 10000 | 8.3 | 326 | 6800 | 140 |  | Bilateral Lower |
| 3 | 71 | 55 | 371 | 7.5 | 57 |  | 0.013 | 3600 | 14 | 158 | 3240 | 295 |  | Bilateral Diffuse |
| 4 | 154 | 59 | 946 | 8.9 | 100 | 1170 | 0.016 | 10000 | 13.9 | 199 | 8100 | 450 | 2.46 | Bilateral Diffuse |
| 5 | 162 | 281 | 1005 | 7.8 | 95 | 1054 | 0.005 | 4800 | 13 | 187 | 3200 | 230 | 2 | Bilateral Diffuse |
| 6 | 42 | 188 | 1317 | 5.5 | 82 | 1072 | 0.2 | 11000 | 1 | 123 | 9800 | 990 | 0.8 | Bilateral Diffuse |
| 7 | 440 | 89 | 1118 | 3 | 61 | 1256 | 0.011 | 13800 | 12.6 | 304 | 12800 | 842 |  | Right Diffuse |
| 8 | 133 | 424 | 1211 | 5.3 | 105 | 988 | 0.01 | 7200 | 12.5 | 269 | 5900 | 720 |  | Bilateral Lower |
| 9 |  | 153 | 901 | 4 | 77 | 1066 | 0.008 | 8200 | 13.5 | 200 | 6725 | 902 | 6.5 | Bilateral Diffuse |
| 10 |  | 138 |  | 7.4 | 104 | 612 | 0.03 | 9700 | 12.6 | 233 | 8827 | 436 |  | Bilateral Lower |
| 11 | 87 | 570 | 2000 | 8 | 58 | 1360 |  | 12000 | 14.5 | 338 | 10300 | 840 | 0.59 | Left Upper |
| 12 | 162 |  |  | 4.6 | 55 | 1127 |  | 8000 | 12 | 19 | 6250 | 960 |  | Bilateral Diffuse |
| 13 |  |  |  | 7 | 75 | 312 |  | 400 | 8.4 | 20 | 7.2 | 356 |  | Left Diffuse |
| 14 | 147 | 1252 | 630 | 7.5 | 132 | 880 | 0.034 | 8600 | 13.5 | 196 | 6700 | 1118 | 1.06 | Right Diffuse |
| 15 | 76 | 325 | 890 | 3.6 | 87 | 1664 |  | 133000 | 9.6 | 241 | 13300 | 106400 | 1.6 | Bilateral Lower |

CRP: C-reactive protein, CK: Creatinine Kinase, Fr: Ferritin, U: Urea, Cr: Serum Creatinine, LDH: Lactate Dehydrogenase, WBC: White Blood Cells, Hb: Hemoglobin, Plt: Platelet, N: Neutrophils, L: Lymphocyte, DD: D-Dimer, FEU: fibrinogen-equivalent units. CXR: Chest X-ray.
